# A retrospective evaluation of an online group ketogenic metabolic therapy intervention on mental health outcomes

**DOI:** 10.3389/fnut.2026.1751564

**Published:** 2026-03-04

**Authors:** Erin L. Bellamy

**Affiliations:** School of Psychology, University of East London, London, United Kingdom

**Keywords:** anxiety, depression, ketogenic diet, ketogenic metabolic therapy, metabolic psychiatry, online program

## Abstract

**Background:**

Conventional treatments for depression and anxiety, including pharmacotherapy and psychotherapy, often fail to achieve long-term symptom remission and are associated with side effects, limited accessibility, and high attrition. Ketogenic metabolic therapy (KMT) has emerged as a potential adjunctive intervention, with studies showing improvements in metabolic and mental health outcomes. However, research on remotely delivered, group-based KMT remains limited. This study evaluates the feasibility of an online, group-based KMT program integrating psychoeducation, professional guidance, and community support on symptoms of depression and anxiety in adults with varying mental health conditions.

**Methods:**

A retrospective evaluation of 19 self-referred participants with baseline PHQ-9 and GAD-7 scores > 4. Participants followed a ketogenic diet tailored to individual macronutrient targets. Depression (PHQ-9) and anxiety (GAD-7) were assessed at baseline, 4, 8, and 12 weeks.

**Results:**

Mean PHQ-9 scores decreased from 13 to 5 over 12 weeks, representing a 62% reduction, with 71% achieving clinically meaningful improvement. Mean GAD-7 scores decreased from 13 to 7, a 46% reduction, with 79% achieving clinically meaningful improvement. Eight participants reached remission for depression and nine for anxiety. Participants achieved blood ketone levels > 0.5 mmol/L 85% of the time, indicating high adherence with mean ketone levels of 1.1 mmol/L. No serious adverse events were reported, and all participants completed the intervention.

**Conclusion:**

This remotely delivered, group-based KMT appears feasible and was associated with clinically meaningful reductions in depression and anxiety over 12 weeks. These findings support the potential of KMT as a scalable, transdiagnostic approach to conventional psychiatric care. Future research should evaluate larger samples and longer-term outcomes.

## 1 Introduction

Treatment for depression includes antidepressant medication, psychotherapy or a combination of these approaches (1, 2), but these conventional approaches often fall short in meeting the diverse needs of those with mental health conditions. Despite being the standard of care, both antidepressant medications and psychotherapies have significant limitations long-term. Research suggests that even though psychotherapies can be more effective than control conditions, over 50% of patients do not respond adequately. Similarly, though antidepressants are widely prescribed and can be effective in acute situations, approximately half of those taking such medications continue to experience significantly impaired quality of life (3, 4).

Many treatments do not address patient-centered outcomes (5), with many individuals finding the psychiatric medications intolerable due to their long-term side effects and ability to cause adverse reactions (5, 6). Alongside this, access to effective treatment remains a substantial barrier. In the UK, the number of individuals waiting for mental health care by 29 has increased % over the past 2 years, with some enduring waiting periods of 2 to 4 years (6–8). In 2024, approximately 1 million people were waiting for mental health care in the UK (9). These long waits are also associated with larger dropouts once treatment has begun, likely due to the long waits between assessment and secondary follow-up (10). This lack of accessible care highlights a substantial gap in mental health services.

In addition, standard treatments like cognitive behavioral therapy (CBT) have shown limited efficacy, particularly in sustainable, long-term recovery (1, 2). For many, these approaches do not adequately address their symptoms or comorbidities, leaving a substantial proportion of individuals without a viable path to recovery. Therefore, there is a need for innovative, scalable, and patient-centered interventions that can be accessed and implemented as soon as an individual seeks support, and that will address these shortcomings and better support sustainable recovery.

Research into ketogenic metabolic therapy (KMT) for mental health conditions is ongoing, with many studies showing positive effects on both physical and mental health outcomes in those with varying levels of depression, anxiety and other psychiatric diagnoses (11–25). Specifically, a recent pilot study in 16 college students with major depressive disorder reported a decrease in depression of 69% as measured by the PHQ-9 over 10–12 weeks with a therapeutic ketogenic diet as an adjunctive therapy to standard care (17). Ketosis was achieved 73% of the time. There appear to be at least six shared mechanistic pathways at play, brain glucose hypometabolism, insulin resistance, neurotransmitter imbalance, oxidative stress, mitochondrial dysfunction and neuroinflammation (26, 27).

These findings are encouraging and offer an additional therapy to trial alongside standard care. The ketogenic diet may also offset the negative side effects of psychiatric medications, and it may improve symptoms independently through the previously mentioned mechanistic pathways. A key limitation in the studies to date is that the intervention is delivered face-to-face, limiting the number of people the therapy can reach. Prior research (14) recommends investigating KMT in a remote care setting, focused on mental health symptoms monitoring, dietary adjustments based on ketone levels and frequent coaching over a period of at least 12 weeks (28). Although individual support is ideal, scalability needs a remote, cost-effective approach to reach individuals both geographically far and wide, as well as those who are unable to leave their homes due to symptom severity.

This study is a retrospective evaluation of an online group ketogenic metabolic therapy intervention on mental health outcomes, primarily depression and anxiety. The intervention included psychoeducation and ongoing professional and community support. There is a need to support individuals to learn and effectively apply the intervention, but also to sustain it long term if they experience improvements in their mental health symptoms and overall wellbeing. Previous studies have ranged from 6 to 16 weeks, with only one study extending up to 248 days (20), suggesting a need for a two-phase care model: (1) education and implementation, and (2) long-term lifestyle support.

### 1.1 Aims

The aim of this study is to evaluate the efficacy of an online group-based ketogenic metabolic therapy intervention, integrating psychoeducation, professional guidance, and community support, on symptoms of depression (PHQ-9) and anxiety (GAD-7) in adults with varying mental health conditions. This study will also assess the feasibility and impact of an education and implementation model for KMT as a first-phase intervention. This will determine whether structured teaching and guided support enable participants to achieve meaningful clinical improvements in mental health outcomes. Positive outcomes may inform the development of a longer-term, two-phase care model.

## 2 Methods

### 2.1 Study design

A retrospective evaluation and analysis were performed to assess the impact of KMT on mental health outcomes after 12 weeks, during a 6-month online intervention using deidentified data from the “IKRT Foundations” group metabolic mental health program.^1^

### 2.2 Participants

This study was an audit of pre-existing routine practice, and participants were self-referred. Participants included paying clients of the “IKRT Foundations” group program. Participants were considered as eligible if they were at least 18 years of age; had scale scores of at least mild levels of depression and anxiety (PHQ-9 > 4, GAD-7 > 4) and had completed at least 8 h of education sessions and provided psychological measure questionnaire responses for baseline, 4, 8, and 12 weeks. If participants were taking psychiatric medication, they must have had professional clinical oversight via a prescriber or psychiatrist. They also must have been willing to perform finger stick blood testing. Participants were non-diabetic, clinically stable (no hospitalization in the past 3 months), not pregnant, nursing or planning to become pregnant, and not taking any incompatible medications (e.g., SGLT2 inhibitors or anticoagulants). Detailed recommendations on medical contraindications were also followed as per previous research trials (29).

A total of 19 participants satisfied all inclusion criteria. Participants had tried many conventional and alternative therapies with some benefit but limited long-term success. Therapies mentioned were; psychotherapy, talk therapy, counseling, meditation, cognitive behavioral therapy (CBT), exercise, ayurveda, acupuncture, psychoanalysis, eye movement desensitization and reprocessing (EMDR), visualization, trauma therapy, internal family systems therapy (IFS), process group therapy, intensive outpatient therapy, transcranial magnetic stimulation (TMS), dialectical behavior therapy (DBT), neurofeedback, ketamine, tanning beds, 12 step groups, orthomolecular approaches, somatic therapy, and mindfulness practices. Some participants continued these alongside the study, but no new therapies were initiated during the intervention period. From a dietary perspective, one participant followed a vegan ketogenic diet, with all other participants following an omnivorous ketogenic diet.

### 2.3 Materials and measures

#### 2.3.1 KMT intervention

Integrative Ketogenic Research and Therapies Ltd. (IKRT) supports individuals to safely and effectively implement KMT through their innovative, scalable model, which bridges the gap between science and real-world application to improve metabolic and mental health. This program has been delivered safely and effectively since its inception in July 2024, and a case study has been recently published on the outcomes (30). This program is designed to work alongside conventional care provided by primary services. This is currently the only program of its kind in the United Kingdom.

The intervention includes personalized macronutrient targets for each participant to track on a food tracker, access to all educational recordings once they have been taught live, protocols to track blood ketones and glucose, how-to videos, educational resources, supplement recommendations, electrolyte recommendations, ketogenic food lists and a supportive private online community that is moderated regularly by the IKRT team. Only those who have completed an IKRT program have access to the online community, which regulates the information shared.

The intervention consisted of 8 h of education delivered over 8 weeks, followed by weekly 1-h support and Q&A sessions for 24 weeks. It was delivered by a practitioner trained in KMT implementation. Psychological assessments were completed at baseline and every 4 weeks for the duration of the 6-month program. Intermittent check-in forms were completed by participants, providing updates and comments on their experience of the program thus far. This retrospective analysis presents data on mental health outcomes collected up to the halfway point of the program, over 12 weeks.

#### 2.3.2 Diet composition

A Moderate Atkin’s Dietary (MAD) approach was utilized, as research shows improved adherence compared to the classic ketogenic diet (31, 32). Diet composition ranged from 1.5:1 to 2:1 ratio per participant, and diets were calorie matched with the participants’ TDEE, depending on their age, gender, activity level, diet preference, and medication intake. Participants were asked to track their macronutrient intake on a food app such as MyFitnessPal or Cronometer, initially as a learning tool and were encouraged to eat ad libitum as per previous studies (14). Participants made their own meals using a ketogenic food list and made sure that their meals met their daily macronutrient targets. It was expected that participants would experience reduced appetite once they reached levels of nutritional ketosis (0.5 mmol/L blood ketone levels).

#### 2.3.3 Psychological measures

Assessments for depression and anxiety were administered at baseline and at multiple time points throughout the intervention. The dependent variables measured through valid psychometric scales at each time point were depression, PHQ-9 (33) and anxiety, GAD-7 (34). All psychological measures have high reliability and validity within the published literature. The Patient Health Questionnaire (PHQ-9) is a diagnostic tool for assessing the severity and presence of depressive symptoms (33). The Generalized Anxiety Scale (GAD-7) is used to determine anxiety severity (34) and identify other anxiety disorders such as panic, social anxiety disorder, and post-traumatic stress disorder (35). These assessments were conducted at baseline, 4, 8, and 12 weeks to track changes over time and were sent to participants automatically and regularly via the IKRT platform, which is HIPAA compliant.

### 2.4 Statistical analysis

Baseline characteristics and demographics were captured and recorded. Descriptive statistics were used to summarize baseline and follow-up data, including means and standard deviations. Statistical analysis was carried out using SPSS V28, and a range of tests were employed on the data to assess normality and variance, such as the Shapiro-Wilk test, Analysis of Variance (ANOVA), and Friedman’s test. An Intent to Treat (ITT) analysis using the “Last Value Carried Forward” approach was also applied to participants who completed the baseline testing and at least one more PHQ-9 and GAD-7 measure over the 12 weeks, in keeping with recent publications (17, 36). The significance level for all tests was set at α ≤ 0.05.

## 3 Results

The majority of the participants were female (*N* = 15) (78.9%), and the rest were male (*N* = 4) (21.1%); no participants identified as another gender. Ages ranged from 26 to 63, with the largest number of participants falling into the female, 45–54 years old age category. Age categories were: 25–34 (*N* = 3) (15%), 35–44 (*N* = 5) (26%), 45–54 (*N* = 7) (37%), and 55–64 (*N* = 4) (21%).

Psychiatric diagnoses varied across participants with some participants having multiple psychiatric diagnoses. These are described as reported by the participants; Generalized Anxiety Disorder (*n* = 14), Bipolar II (*n* = 5), Depression (*n* = 5), ADHD Inattentive (*n* = 3), Bipolar I (*n* = 2), Major Depression (*n* = 1), Severe Depression (*n* = 1), Recurrent Depressive Disorder (*n* = 1), Recurrent Major Depressive Disorder (*n* = 1), Social Anxiety (*n* = 1), Seasonal Affective Disorder (*n* = 1), Autism (*n* = 1), Combined Type ADHD (*n* = 1), Paranoid Schizophrenia (*n* = 1), Unspecified Schizophrenia F20.9 (*n* = 1), and Premenstrual Dysphoric Disorder (PMDD) (*n* = 1). Seven participants had previously been admitted to inpatient psychiatric units, with five participants stating multiple admissions, between 3 and 10.

Five participants were not taking any medications, while the remaining 14 participants were taking between 1 and 5 medications each. A total of 12 participants were taking prescribed psychiatric medications with limited effect. Psychiatric medications were; Vortioxetine (*n* = 3), Lamotrigine (*n* = 3), Bupropion (*n* = 3), Clonazepam (*n* = 2), Methylphenidate (*n* = 2), Seroquel (*n* = 1), Oxcarbazepine (*n* = 1) Prozac (*n* = 1), Vyvanse (*n* = 1), Escitalopram (*n* = 1), Buspirone (*n* = 1), Aripiprazole (*n* = 1), Brexpiprazole (*n* = 1), MAOI tranylcypromine (*n* = 1), Lithium (*n* = 1), Duloxetine (*n* = 1), Naltrexone (*n* = 1), Trazadone (*n* = 1), Hydroxyzine (*n* = 1), Quetiapine (*n* = 1), Mirtazipine (*n* = 1), and Elvanse (*n* = 1).

Five participants were taking medications for their physical health: Estradiol (*n* = 3), Levothyroxine (*n* = 3), Progesterone (*n* = 2), Testosterone (*n* = 1), Tamsulosin (*n* = 1), Colesevelam (*n* = 1), Meloxicam (*n* = 1), NP Thyroid (*n* = 1), Liothyronine (*n* = 1), and Remicade (*n* = 1).

In the month prior to starting the intervention, three participants had adjustments to their medications; reduction in Lamotrigine and stopped Lurasidone (*n* = 1), reduction in Vortioxetine (*n* = 1) and increase in Levothyroxine (*n* = 1). No medication changes were made during the 12-week intervention.

### 3.1 Adverse side effects during transition to diet

Subjective reports of adverse effects during transition to the diet were communicated through the weekly check-in forms and discussed during the weekly Q&A calls. Reported symptoms included mild irritability, jitteriness, fatigue, headache, and difficulty falling asleep. All resolved within 2 weeks with dietary guidance. Education and ongoing support were provided throughout the transition, and no significant or persistent adverse effects were reported.

### 3.2 Adherence

Two participants out of 19 were removed from the PHQ-9 dataset as their baseline PHQ-9 scores were already within the “normal range” of 4 points or less. No participants were removed from the GAD-7 dataset. All participants stayed for the duration of the 12-week intervention despite two participants experiencing significant negative life events. The data from these participants has not been removed to reflect real-world clinical scenarios and outcomes.

### 3.3 Normality

Normality of variables was assessed using the Shapiro-Wilk test with α ≤ 0.05. The normality of depression variables at baseline was normally distributed (*p* > 0.05), and therefore, a one-way repeated measures ANOVA was applied. For anxiety, the Shapiro-Wilk test showed a significant departure from normality at baseline, *W*(19) = 0.90, *p* = 0.04, suggesting that the data were not normally distributed (*p* < 0.05). Therefore, the non-parametric Friedman test was applied.

### 3.4 Psychological outcome measures

#### 3.4.1 Depression

**FIGURE 1:**
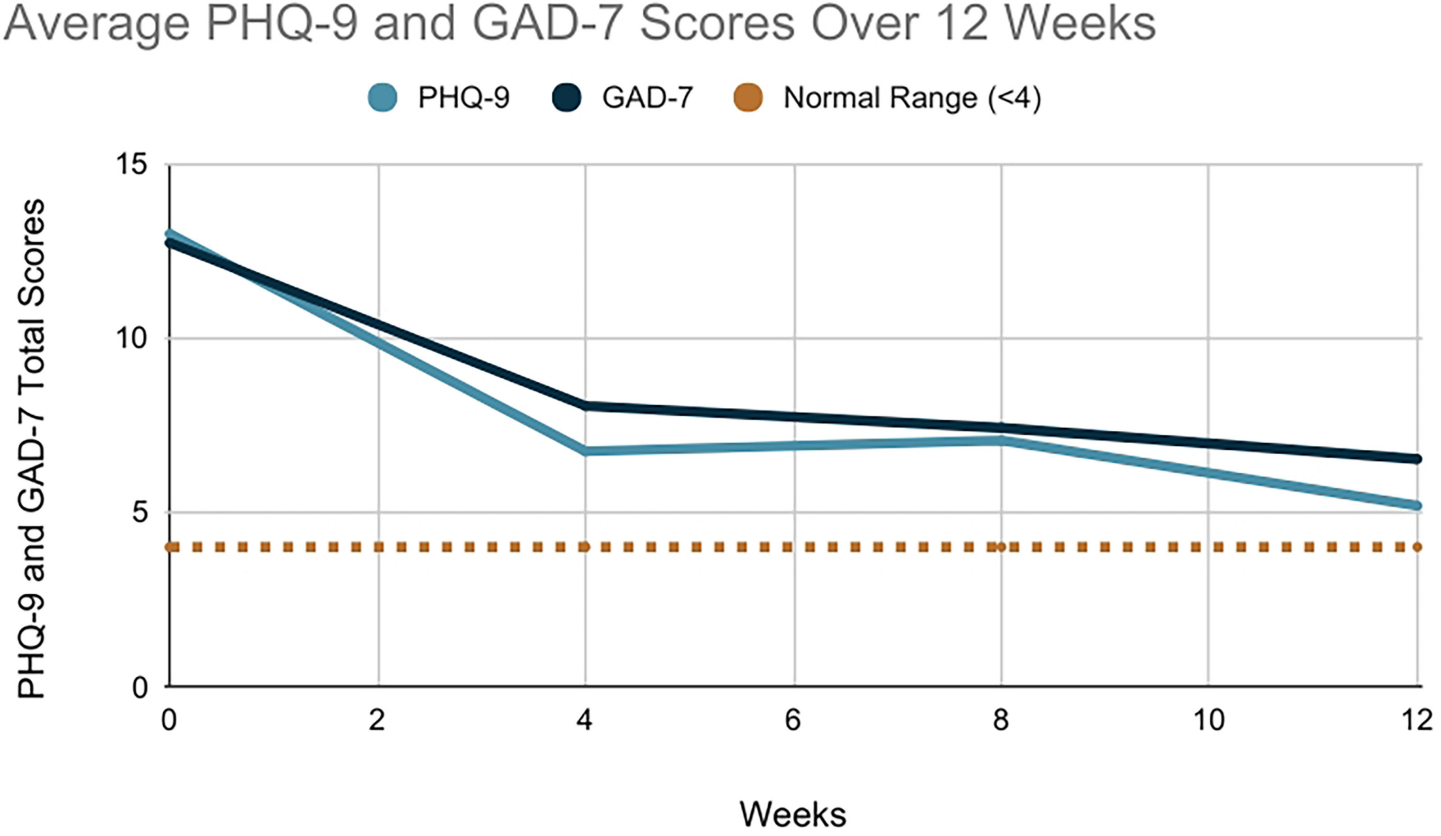
Average reduction in Patient Health Questionnaire (PHQ-9) and Generalized Anxiety Disorder (GAD-7) total scores over 12 weeks.

For the PHQ-9 data, there were 17 participants to report on. At baseline, the mean PHQ-9 score was 13 (SD = 7), indicating moderate levels of depression. Scores across participants varied, with the highest individual score of 27 (severe) and the lowest of 5 (mild). From baseline to 12 weeks, the mean reduction in PHQ-9 scores was eight points, representing a clinically meaningful improvement in depression (≥5 point reduction). Depression scores decreased by 62% on average. A total of 15 out of 17 participants (88%) experienced an improvement at some point during the intervention. A total of 12 participants (71%) experienced a clinically meaningful improvement in their depressive symptoms with at least a drop of five points on the PHQ-9 (Figure 1).

**FIGURE 2:**
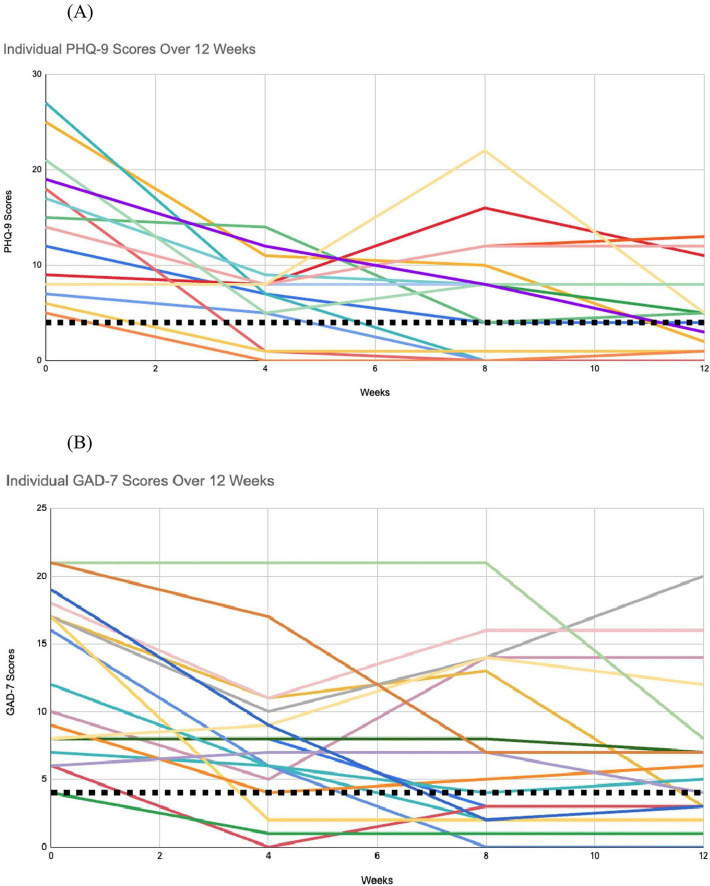
Individual reduction in PHQ-9 and GAD-7 total scores over 12 weeks. **(A)** Patient Health Questionnaire (PHQ-9). **(B)** Generalized Anxiety Disorder (GAD-7).

Four participants experienced worsening of symptoms at some point during the intervention. One participant experienced a clinically meaningful improvement at week 4, before experiencing worsening of symptoms at week 8, which were maintained at week 12. Another participant experienced an improvement in depressive symptoms at week 4 before experiencing an increase in symptoms at week 8, followed by a reduction at week 12, in a zig-zag fashion. No explanation was available for these results. For two other participants, one experienced worsening of depressive symptoms from week 4 until week 12, and another participant experienced a worsening of symptoms from week 4 to week 8, followed by a clinically meaningful reduction in symptoms by week 12. These changes were due to significant unforeseen life events. These are identifiable in Figure 2.

A paired samples *t*-test was conducted to compare mean depressive symptom scores at baseline and 12 weeks, to see if there was a difference between the two. There was a statistically significant difference in the scores from baseline (*M* = 13.4, SD = 6.8) to the end of intervention at week 12 (*M* = 5.1, SD = 4.3); *t*(16) = 3.91, *p* = 0.001, suggesting that the decrease in depressive symptom scores was due to the intervention. A one-way repeated measures ANOVA was carried out to assess the change in mean depressive symptom scores across the intervention and to see if there was a significant change across the three time points. Mauchly’s test indicate that the assumption of sphericity had been violated, χ^2^ (5) = 17.78, *p* = 0.003, and therefore degrees of freedom were corrected using Greenhouse-Geisser estimates of sphericity (ε = 0.56) The effect of the intervention on depression scores was significant at the α ≤ 0.05, *F*(1.67, 26.7) = 9.11, *p* = 0.002, partial η^2^ = 0.363. This indicates a strong treatment effect from the intervention, consistent with a clinically meaningful improvement in PHQ-9 scores over time.

To see where the differences occurred, *post hoc* pairwise comparisons with a Bonferroni adjustment were carried out. Results indicated that there was no significant difference between the depression scores at baseline and 8 weeks, (*p* = 0.11), at 4 and 8 weeks, (*p* = 1.0), at 4 and 12 weeks, (*p* = 0.55) and at 8 and 12 weeks, (*p* = 0.51). Compared to baseline, depression scores were significantly lower at 4 and 12 weeks (*p* < 0.05).

#### 3.4.2 Anxiety

For the GAD-7 data, all 19 participants were reported on. At baseline, the mean GAD-7 score was also 13 (SD = 5.7), indicating moderate levels of anxiety. Scores varied across participants, with the highest individual score of 21 (severe) and the lowest of 4 (minimal). From baseline to 12 weeks, the mean reduction in GAD-7 scores was six points, representing a clinically meaningful improvement in anxiety (≥4 point reduction). Anxiety scores decreased by 46% on average (Figure 1).

All participants experienced an improvement at some point during the intervention. A total of 15 participants (79%) experienced a clinically meaningful improvement in their anxiety with at least a drop of four points on the GAD-7. As was reported in the depression results, the same four participants experienced worsening of symptoms at some point during the intervention. Two participants experienced a clinically meaningful improvement in their anxiety scores at week 4, before experiencing a worsening of symptoms at week 8, which was maintained at week 12. No explanation was available. Two participants experienced worsening of anxiety symptoms from week 4 and week 8, respectively, due to significant unforeseen life events. These are identifiable in Figure 2.

A Wilcoxon signed-rank test was carried out to determine if there was a difference across time points. The results indicated that anxiety scores were significantly lower at 12 weeks (mean rank = 7.8) compared to baseline (mean rank = 10.4), *z* = −2.88, *p* = 0.004. A Friedman test was carried out to assess the change in mean anxiety scores across the intervention and to determine if there was a significant change across the three time points. The test revealed a significant effect of time on anxiety scores, χ^2^ (3) = 16.07, *n* = 19, *p* < 0.001, with a small effect size indicated by Kendall’s *W* = 0.282.

#### 3.4.3 Ketone data

**FIGURE 3:**
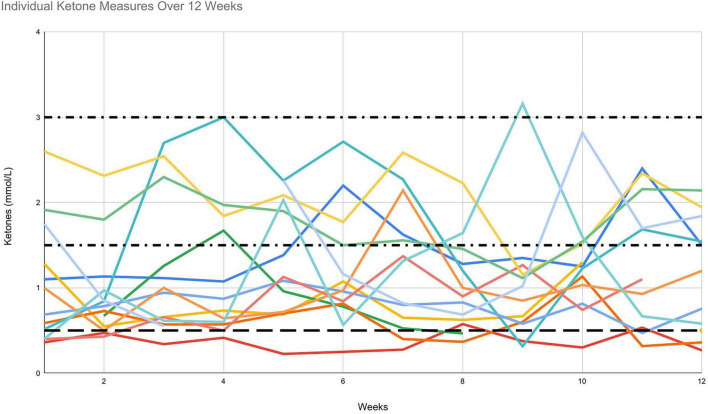
Individual blood ketone measurements over 12 weeks.

**FIGURE 4:**
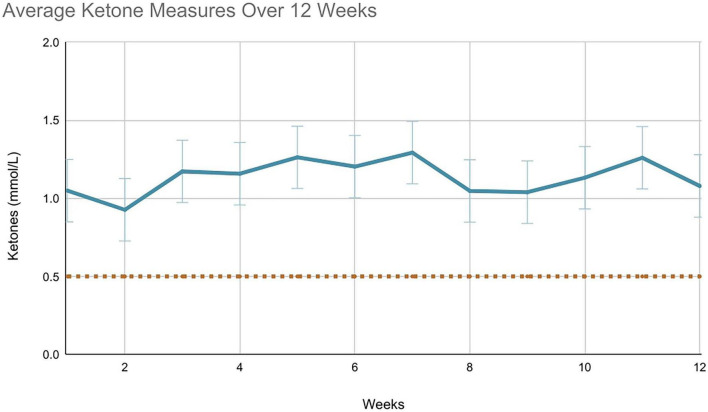
Average blood ketone measurements over 12 weeks.

Daily blood ketone levels were available for 15 out of 19 participants over the 12 weeks. Daily (morning fasted) ketone reading completion varied from 62% to 100%. On average, participants completed daily ketone testing 81% of the time over the 12 weeks. Participants tracked beta-hydroxybutyrate levels using the Keto-Mojo^®^ GK+ blood glucose and β-ketone dual monitoring system (Figures 3, 4). Of the 15 participants, 10 were fully adherent with ketone levels > 0.5 mmol/L > 80% of the time, two were semi-adherent, 60%–80% of the time, and one was non-adherent, with suitable ketone levels < 50% of the time. This stratified reporting is in keeping with other studies (21, 37). The average daily (morning) blood ketone reading was 1.1 mmol/L (SD = 0.5), similar to that reported in the literature (23).

## 4 Discussion

For this study, 19 participants took part, which is similar to other studies in this area whose sample ranged from 1 to 31 (13, 17, 20, 21, 23, 30, 38, 39). In summary, regardless of baseline scores, all participants (100%) reported a decrease in anxiety, and 88% of participants reported a decrease in depression at some point over the intervention. Eight participants (47%) reached remission from depression (PHQ-9 < 5), and nine participants (47%) reached remission from anxiety (GAD-7 ≤ 5). These findings report that, in participants with varying diagnoses, a reduction in both PHQ-9 (*n* = 19) and GAD-7 (*n* = 17) scores was demonstrated over 12 weeks, with a mean decrease of eight points on the PHQ-9 and six points on the GAD-7. This suggests a substantial reduction in both depression and anxiety symptoms. On average, depression scores decreased by 62% and anxiety scores decreased by 46%.

Given that reductions of five points on the PHQ-9 (33) and four points on the GAD-7 (34) are considered clinically significant, these outcomes suggest meaningful clinical improvements. These findings are consistent with previous reports in the literature (12, 13, 17, 20, 21, 23, 30, 40). Some of those who experienced an increase in depression and anxiety scores experienced extreme life events that contributed to their results, suggesting that though KMT and ketosis appear to provide stability, scores will vary from time to time, in line with the “normal” human experience. Others may have needed more time to adapt to the intervention, and future follow-up studies and studies of longer durations would allow for this initial adaptation period. The reduction in depression is similar to a recent pilot study that reported a decrease of 69% as measured by the PHQ-9 over the same timeframe (17). However, this study was carried out online in a group format, whereas the pilot study was in person. With similar improvements seen in this study, it may be worth looking at online group programs in order to scale and reach more people with KMT.

Participants were in ketosis (>0.5 mmol/L) 85% of the time, which is in keeping with other reports that range from 73% to 100% (12, 13, 17, 23, 30). The mean morning ketone level was 1.1 mmol/L. Interpreting a potential dose-response relationship between ketone levels and symptom improvement is limited as readings were taken only once per day. As ketone levels are typically at their lowest point in the morning (41), and the mean morning ketone reading was 1.1 mmol/L within the range of “optimal ketosis,” this suggests participants were likely in “optimal ketosis” for most of the day (23). Future research should employ the use of continuous ketone monitors to assess this further.

### 4.1 Strengths, limitations and future research

These improvements were achieved with self-selecting participants in a paid program that was fully remote and delivered in a group setting. None of the participants dropped out over the duration of the intervention compared to recent studies that reported attrition rates of 8%–33% (17, 20, 21, 23). The high retention rate is likely due to the structure of the program, psychoeducation, professional guidance and ongoing support. Participants prepared all of their own meals, despite many beginning the program with high levels of depression and anxiety. No ready-made ketogenic meal services were used. Instead, participants followed a structured food list, and once ready, progressed to tracking macronutrients using a food app. This approach supported autonomy and personal food preferences and promoted the sustainability of the diet. These findings suggest that the intervention is both feasible and effective, although further studies are needed to rigorously evaluate its effectiveness.

This study has several limitations. Selection bias is likely as participants sought KMT to support their mental health and may not represent the wider psychiatric population. Many had tried multiple medications and therapies with limited benefit, and therefore, this group may reflect a more chronic subgroup. The sample size was small (*n* = 19), which limits generalisability. Only depression and anxiety outcomes are reported here. Other areas of mental health are likely to have changed but were not captured. Without a control arm, natural symptom fluctuations cannot be ruled out, however, the inclusion of objective biomarkers (blood ketone levels) and validated psychiatric assessments helps mitigate this concern. As a real-world intervention, outcomes may also have been influenced by uncontrolled factors such as socioeconomic circumstances, food affordability, kitchen access, psychological stressors, health literacy and availability of mental health service appointments. These barriers are important to recognize when interpreting the results and considering the scalability of this therapeutic model.

The successful implementation of this fully remote, group based KMT program demonstrates a potentially scalable and accessible model for underserved or geographically dispersed populations. If expanded, it may help improve clinical outcomes when used alongside standard care.

Future research should include a larger pilot trial delivered over the full 6-month program to assess longer-term outcomes and adherence. Additional psychological and functional measures should also be collected to provide a more comprehensive understanding of change over time. If future results are positive, the next step will be to evaluate how this intervention can be integrated into existing healthcare and mental health systems at scale.

### 4.2 Conclusion

This retrospective evaluation suggests that a remotely delivered, group-based KMT intervention can lead to significant improvements in depression and anxiety over 12 weeks. Participants demonstrated high adherence with clinically relevant reductions in PHQ-9 and GAD-7 scores, suggesting feasibility in a real-world setting. The findings from this study further support the potential effectiveness of a transdiagnostic treatment model for individuals with varying psychiatric diagnoses. Although limited by the lack of a control group, the findings align with emerging evidence supporting metabolic therapies as a promising adjunct to psychiatric care. Further controlled research with larger samples, longer follow-up, and broader outcome measures is needed to confirm these effects and to determine how KMT can be integrated into scalable models of mental healthcare.

## Data Availability

The raw data supporting the conclusions of this article will be made available by the authors, with all participant information fully de-identified to protect privacy and in compliance with HIPAA standards upon reasonable request. Requests to access the datasets should be directed to e.l.bellamy@uel.ac.uk.

## Footnotes

1
www.ikrt.org
